# Supplementary material for: MYC Causes Multiple Myeloma Progression via Attenuating TP53-Induced MicroRNA-34 Expression
Source: Genes (Basel). 2022 Dec 29;14(1):100. doi: 10.3390/genes14010100 (PMC9859619; doi:10.3390/genes14010100)
Supplement: Supplementary file 1 [file genes-14-00100-s001.zip › genes-1878063-supplementary.pdf]

Supplementary Results

P53 protein accumulation and p53 overexpression upregulated primary and mature miR-34 in human multiple myeloma cell lines (HMCLs)

Western blot analysis verified increased p53 protein in MM.1S, KMS27 but not changed in KMS28BM, KMS26 and OPM2 by the treatment with nutlin-3. p53 protein was not detected in KMS11 **(Figure S1A)**.

A

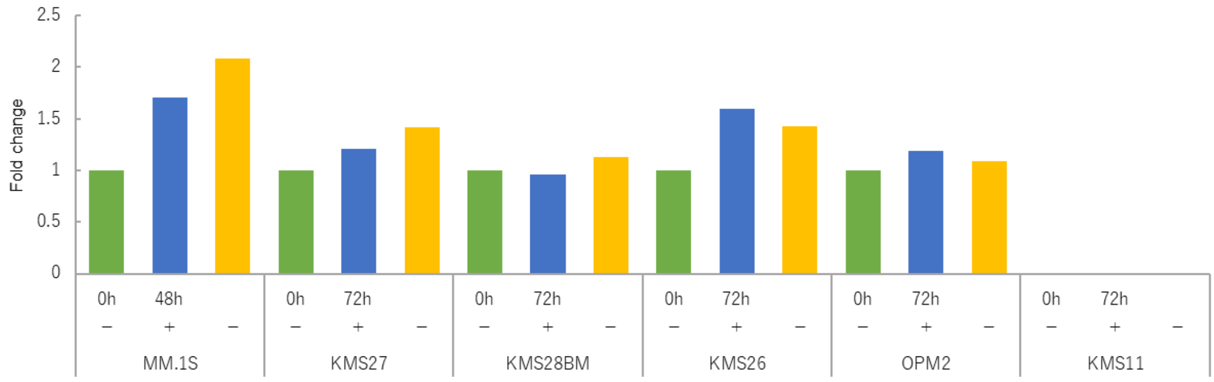

Figure S1. Changes in p53 protein expression in response to nutlin-3 in six myeloma cell lines. MM.1S was treated for 0, 48 h with nutlin-3 at concentrations of 0 µM and 1 µM. KMS27, KMS28BM, KMS26, OPM2 and KMS11 were treated for 0, 72 h with nutlin-3 at 0 µM and 10 µM. Green, for 0h with 0µM nutlin-3; Blue, for 48 or 72 h with 0 µM nutlin-3; yellow, for 48 or 72 h with 1 or 10 µM nutlin-3. ND: Not Detected.

Osteosarcoma cell line and HMCLs proliferation after treatment with MDM2 inhibitor nutlin-3

In TP53 wild-type cell line U2OS and MM.1S, nutlin-3 markedly reduced cell proliferation **(Figure S2A, B)**. For those cell lines, 10µM nutlin-3 were too toxic and resulted in cell death at 72h. In KMS27, KMS28BM, KMS26, OPM2, and KMS11, proliferation was not significantly increased by 10µM nutlin-3 **(Figure S2C-G)**.

A U2OS

B MM.1S

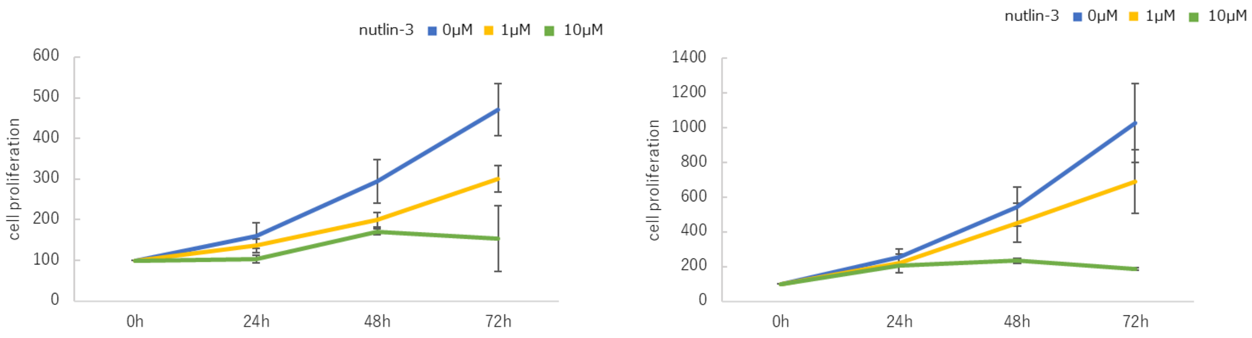

C KMS27

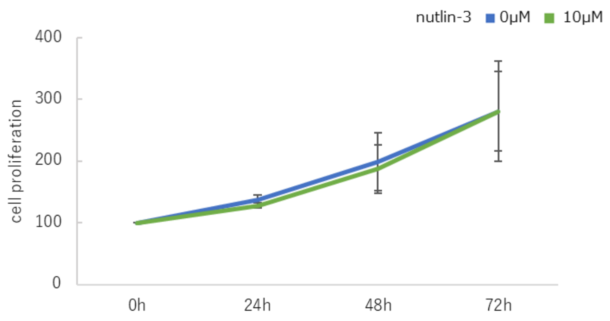

D KMS28BM

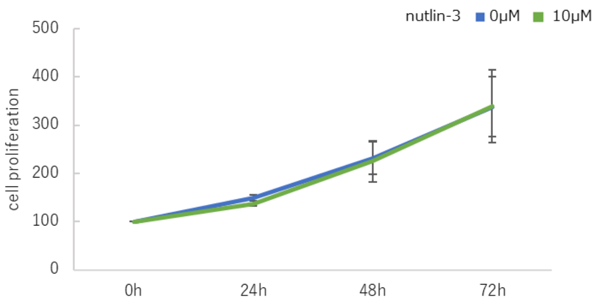

E KMS26

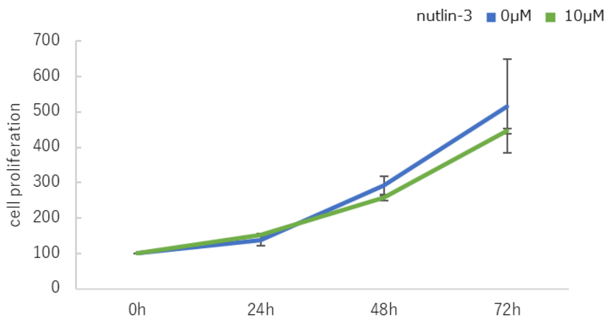

F OPM2

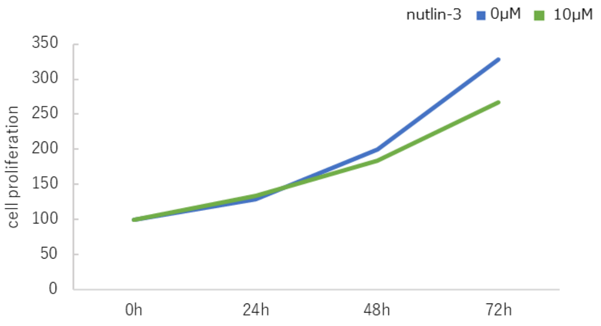

G KMS11

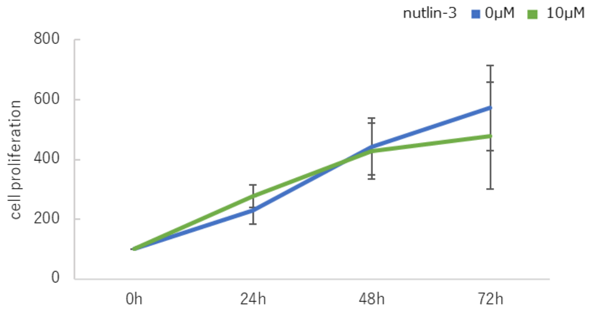

Figure S2. Effects of nutlin-3 for cell proliferation. (A) U2OS, (B) MM.1S, (C) KMS27, (D) KMS28BM, (E) KMS26, (F) OPM2 and (G) KMS11 were treated for 0h-72h with nutlin-3 0μM, 1μM, 10μM. Only experiments with OPM2 were performed twice. Other experiments were performed in triplicate. Error bars show the standard deviation (SD) across those experiments.Blue line, 0μM; yellow line, 1 μM; green line, 10 μM.
